# Optimal botulinum toxin therapy of dystonia in Germany: what would it cost?

**DOI:** 10.1007/s00702-024-02845-4

**Published:** 2024-12-24

**Authors:** Dirk Dressler, Eckart Altenmüller, Lizhen Pan, Fereshte Adib Saberi

**Affiliations:** 1 Movement Disorders Section, Department of Neurology, Hannover Medical School, Carl-Neuberg-Str. 1, D-30625 Hannover, Germany; 2 Neurotoxin Research Center, Tongji University Medical School, Shanghai, China; 3 Institute of Music Physiology and Musicians’ Medicine, University of Music, Drama and Media, Hannover, Germany; 4 Neurotoxin Research Center, Department of Neurology, Tongji University School of Medicine, Shanghai, China; 5 IAB - Interdiciplinary Working Group for Movement Disorders, Hamburg, Germany

**Keywords:** Botulinum toxin, Therapy, Dystonia, Treatment costs

## Abstract

Botulinum toxin (BT) therapy is the therapy of choice for most forms of dystonia. We want to describe its costs, if all dystonia patients in Germany would have access to optimal BT therapy. For this, we combined the latest data on epidemiology of dystonia and dosing of BT therapy for dystonia. Missing data were generated for this study. Based on official German pharmacy sales prices, optimal treatment for all dystonia patients in Germany with a population of 84.1 million would generate annual drug costs of €155.5 million (cervical dystonia 89.3, tardive dystonia 22.1, generalised dystonia 17.9, blepharospasm 9.3, segmental dystonia 5.9, writer’s cramp 5.3, arm dystonia 3.2, oromandibular dystonia 2.3, musician’s dystonia 0.3, spasmodic dysphonia 0.1) This is €1.85 annually per capita or 0.3% of the total 2021 drug budget and 42% of the 2021 Parkinson drug budget of German public insurance companies. Actual costs for the health care system are considerably lower, as there are various discounts to consider. Further reductions would be possible. BT therapy’s individual costs are high. Its costs for the health care system, however, are marginal. Comparing projected and actual costs, would allow estimating availability and quality of BT therapy of dystonia in Germany.

## Introduction

Botulinum toxin (BT) therapy is the therapy of choice for most forms of dystonia. Due to lack of alternative treatment options, its advent has changed therapy for these patients often dramatically. Prices per individual treatment sessions, however, are high. When daily treatment costs are considered, these prices look less dramatic, but treating cervical dystonia with BT still generates annual drug costs of approximately €4000 per patient. These costs look challenging to health care systems. We wanted to explore the costs of BT therapy of dystonia in Germany combining recently published epidemiological and dosing data generated by our institution. These costs are projections based on the assumption, that all patients would have access to BT therapy and that BT therapy’s application is optimal.

## Methods

### Design

This study is a national German cost analysis of BT therapy of dystonia based on its optimal accessibility and its optimal application. The study combines recently published data on epidemiology and on BT dosing in dystonia, both generated by our institution. Missing data were generated as part of this study.

### Epidemiological data

Epidemiological data on the prevalence of dystonia were recently published (Dressler et al. 2022). They include calculations on the total number of patients affected in Germany. Additional data on the national prevalence of musician’s dystonia were generated as part of this study.

### BT dosing data

Data on dosing of BT therapy in dystonia were also recently published (Dressler et al. 2021). They are consensus recommendations for optimal application of BT therapy. Missing data on dosing of BT therapy in tardive dystonia, generalised dystonia and segmental dystonia were retrieved from our computerised data base as part of this study. Data on BT dosing in musician’s cramp were provided by one of the authors (EA). Data on BT dosing in spasmodic dysphonia were reconstructed from previous publications (French et al. 2020).

Dosing data refer to onabotulinumtoxinA (ONA, Botox^®^, Abbvie-Allergan, Wiesbaden, Germany) and incobotulinumtoxinA (INCO, Xeomin^®^, Merz Pharmaceuticals, Frankfurt/M, Germany). To combine dosing data from ONA and INCO, a conversion ratio of 1:1 was applied (Dressler 2009; Dressler et al. 2012, 2014, 2019; Pan et al. 2019). BT therapy using abobotulinumtoxinA was not included in this study, as conversion ratios with other BT drugs are still a matter of discussion.

### Calculations

Pricing of the ONA 100MU standard vial and the XEO 100MU standard vial were evaluated in Gelbe Liste Pharmindex (https://www.gelbe-liste.de) on October 28th 2021. This price reflects the official published pharmacy price, when the drug reaches the patient. According to German taxation, it includes the full Value Added Tax at a current rate of 19%. ONA prices and INCO prices were similar. The mean of both prices ($\text{\euro}$417.08) was used for further calculations. For each patient, four BT injections series per year were assumed, reflecting typical inter-injection intervals of three months.

As a reference, the 2021 total drug budget for German public insurance companies was identified as €46.6 billion (GKV-Spitzenberband 2022), whereas the 2021 Parkinson drug budget for German public insurance companies was €368 million (Arzneimittel-Atlas 2022). Public insurance companies cover about 90% of the total health care insurance market in Germany. 10% are covered by by private insurance companies.

The current of population of Germany is estimated to be 84.1 million (D-Statis 2022).

## Results

### Dosing

Table 1 shows the BT dosing for all major dystonia manifestations. Dosing for cervical dystonia, blepharospasm, writer’s cramp, oromandibular dystonia and arm dystonia originated from one of our previous publications (Dressler et al. 2021). Dosing data on tardive dystonia (428.0 ± 190.6MU, 10 patients), segmental dystonia (208.4 ± 64.6MU, 10 patients) and generalised dystonia (1496.0 ± 327.3MU, 10 patients) were retrieved from our departmental BT database. Dosing data on musician’s dystonia originated from a random sample of 233 patients with musician’s dystonia (198 males, 35 females) treated by EA. Their total BT dose was 28.0 ± 8.9MU per patient per injection series.

**Table 1:** Epidemiology and botulinum toxin therapy of dystonia and cost calculations. Data are based on Dressler et al. 2021 and Dressler et al. 2022

Form of dystonia	Prevalence in Germany [*n*]	Mean BT dose [MU/injection series/patient]	Annual BT usage [MioMU/y]	Annual BT costs [Mio€/y]
Cervical dystonia	20,387	262.6 ± 141.6	21.41	89.3
Blepharospasm	7105	78.8 ± 31.6	2.24	9.3
Writer’s cramp	4479	70.3 ± 55.3	1.26	5.3
Tardive dystonia	3089	428.0 ± 190.6^2^	5.29	22.1
Generalised dystonia	2162	496.0 ± 327.3^2^	4.29	17.9
Spasmodic dysphonia	2008	2.4^1^	0.02	0.1
Segmental dystonia	1699	208.4 ± 64.6^2^	1.42	5.9
Arm dystonia	1236	156.0 ± 143.3	0.77	3.2
Oromandibular dystonia	1081	127.5 ± 69.9	0.55	2.3
Musician’s dystonia	600^3^	28.0 ± 8.9^3^	0.07	0.3
**Total**	**43,846**		**37.3**	**155.5**

Means are given in mean ± standard deviation

*Mio* million; *MU* mouse unit; *y* year; *n* number

^1^Data on spasmodic dysphonia are calculated from French et al. 2020

^2^Data on dosing of botulinum toxin therapy for tardive dystonia, generalised dystonia and segmental dystonia were generated for this study

^3^Data on epidemiology and botulinum toxin therapy of musician’s dystonia were generated for this study

Data on BT dosing in spasmodic dysphonia were calculated from data previously published in the literature. In a recent long-term study on the BT dosing in 126 patients with spasmodic dysphonia, 101 patients were treated bilaterally with ONA 1.28 ± 0.41MU per side and 25 patients were treated unilaterally with ONA1.65 ± 0.62MU per side and per injection series (French et al. 2020). That indicates an average BT total dose of ONA 2.38MU per patient per injection series. In another study, 13 patients were treated unilaterally or bilaterally with an average total dose of ONA 3.9MU (Holden et al. 2007). For our calculations, we will further refer to a BT total dose of 2.38MU per patient per injection series.

### Epidemiology

Epidemiology data originated from one of our previous publications (Dressler et al. 2022) originating from the Hannover Dystonia Registry. Data from this registry refer to a unique reference area with maximal dystonia awareness and maximal dystonia treatment infrastructure. Approximately 85% of dystonia patients identified in this study received BT therapy, either alone or in combination with other treatment modalities.

Data on the epidemiology of musician’s dystonia were re-calculated, as the Hannover reference area are was not typical for the normal German population because of its extremely high concentration of musicians at the University of Music, Drama and Media and in several large professional orchestras. In November 2021, one of the authors (EA), therefore, performed an informal survey in all main BT treatment centres in Germany including Hannover with 70% of all patients, Berlin with 15% of all patients, Düsseldorf with 10% of all patients and Dresden with 5% of all patients. It generated a number of 600 patients with musician’s dystonia receiving BT therapy.

### Prices

Table 2 shows the results of the price evaluation for ONA and INCO. Prices vary considerably between therapeutic and aesthetic BT preparations. Also, prices are influenced by the packaging size. The price for the ONA 100MU therapeutic standard vial was €427.91, for the INCO 100MU therapeutic standard vial it was €406.25. The mean of €417.08 was used in all further calculations.

**Table 2:** Official pharmacy sales price for onabotulinumtoxinA and incobotuolinumtoxinA preparations in Germany inclusive of 19% value added tax

	50MU	100MU	200MU
**onabotulinumtoxinA**			
Botox^®^	*n* = 1: €219.47	*n* = 1: €427.91 *n* = 2: €844.79 *n* = 3: €1261.67	*n* = 1: €844.77
Vistabel^®^	*n* = 1: €127.95	*n* = 1: €231.70	
**incobotulinumtoxinA**			
Xeomin^®^	*n* = 1: €208.15	*n* = 1: €406.85 *n* = 2: €793.79 *n* = 3: €1158.44 *n* = 4: €1540.51	*n* = 1: €796.81 *n* = 2: €1546.24
Bocouture^®^	*n* = 1: €194.04 *n* = 2: €359.51 *n* = 6: €689.84	*n* = 1: €317.10 *n* = 6: €1.343.75	

*n* number of units per package unit

## Discussion

There have been several attempts to produce cost estimates for BT therapy. However, they do not reflect the current practise of BT therapy, especially recent improvements of BT therapy, such as the the high dose therapy and the short interval therapy. They do not consider the entire spectrum of dystonia indications, they are restricted to oligocentric designs and small patient numbers and do not connect them with current epidemiological data (Dodel et al. 1997; Gudex et al. 1997; Brefel-Courbon et al. 2000; Burbaud et al. 2011). This is, to our knowledge, the first study combining current epidemiological data with state of the art dosing data to project maximal costs of optimal BT therapy of dystonia.

### Usage

Our data indicate a usage of 37.3 million MU per year to treat dystonia with optimal BT therapy. BT therapy is indicated in focal dystonia (Dressler et al. 2016). It may also be indicated in patients with more wide-spread dystonia, such as segmental or generalised dystonia, when it may be combined with deep brain stimulation (Dressler et al. 2016). Except for psychogenic dystonia, BT therapy was used in approximately 85% of all of our patients with dystonia (Dressler et al. 2022). Of course, indication of BT therapy in patients with very mild dystonia or with non-focal dystonia is debatable in the individual patient. Dystonia treatment with oral drugs alone has very rarely been sufficient. The actual usage of BT may also be influenced by the treatment algorithms applied, i.e. the dosing per target muscle, the number of target muscles used and the frequency of applications. Current consensus guidelines on BT therapy (Dressler et al. 2021) suggest higher total doses are mainly arising from larger number of target muscles used than previously suggested. Inter-injection intervals may also be reduced to 8 weeks, where necessary (Dressler et al. 2021). Personal experience suggests, that these improved treatment algorithms have not yet been generally adopted. Current treatment practise, therefore, may not always reflect optimal BT therapy.

### Prices

Drug prices in Germany are intransparent. They vary considerably depending on various factors. Prices quoted in this study refer to published pharmacy sales prices. They mark the upper end of the price range. They are only paid by private insurance companies or by patients without insurance. We used these prices, as it is very difficult to estimate the various discount schemes.

In Germany, public insurance companies (Gesetzliche Krankenkassen, GKV) reduce drug prices, either upfront or by enforcing discounted re-importation schemes from low-price countries. Prices for BT drugs applied within hospitals can basically be negotiated between manufacturers and hospitals and distribution charges for pharmacies can be bypassed.

Prices of BT drugs further differ depending on their labelling. As shown in Table 2, BT drugs labelled for aesthetic use (Botox Cosmetic^®^/Vistabel^®^, Bocouture^®^) are lower than the BT drugs labelled for therapeutic use (Botox^®^, Xeomin^®^), although they are identical. Reasons for that are unclear, but probably reflect increased competition and price pressure in the aesthetic market. Also shown in Table 2, prices also vary depending on their packaging: Vials containing larger amounts of BT are usually cheaper than vials containing smaller amounts of BT. Boxes containing several vials are usually cheaper than boxes containing fewer vials. On the other hand, availability of more appropriate vial sizes, may reduce waste and thus may reduce costs (Dressler and Adib Saberi 2017).

### Costs

Based on maximal BT prices not considering various ways of cost reductions, our data suggest, that optimal BT therapy of dystonia applied to all patients in need would require 37.3 million MU of BT annually at a cost of €155.5 million annually for the entire country of Germany with a population of 84.1 million. This would be €1.85 per capita annually or 0.3% of the total 2021 drug budget for German public insurance companies of €46.6 billion (GKV-Spitzenverband 2022). Also, it would be 42% of the 2021 Parkinson drug budget for German public insurers of €368 million (Arzneimittel-Atlas 2022). If the private insurance sector, covering about 10% of the German population, would be included in this calculation, the proportions of the drug budgets would be even lower.

The costs of BT therapy of dystonia for the health care system are also influenced by its regulatory framework. Unlike in many other countries, drug prices in Germany bear the full Value Added Tax of currently 19%. This taxation is included in our calculations. In countries without this taxation, the costs for BT therapy of dystonia would be substantially lower. Prescribing regulations in Germany also affect the actual price of BT therapy. For example, it is not allowed in Germany to share vials amongst patients, even if all possible sterility precautions would be followed. Of course, this regulation generates large quantities of waste, which could be immediately reduced without this regulation. Regulations on the expiry date of reconstituted BT drugs also influence the economics of BT therapy. Recent studies show that reconstituted BT drugs are much are much longer stable than the expiry date suggests (Dressler and Bigalke 2017). Using this information would allow to share reconstituted BT drugs between patients or treatment sessions.

The general level of drug prices in Germany is amongst the highest in the world (Peter and Peterson Foundation 2022). This is also true for BT drugs. Even, if differences of the buying power in different countries is considered, BT drug prices are far from equal. Lower BT prices elsewhere is one of the reasons for busy trans-border traffic of drugs as part of re-importation schemes. Most of all, arising new competition from manufacturers outside of Europe and North America may reduce BT drug costs in the future (Dressler and Johnson 2022).

### Outlook

BT therapy has dramatically improved the treatment situation of patients with dystonia. Although its individual costs seem high, comparing them with the national drug budget show, that they only reflect a tiny proportion of it. Making optimal BT therapy available to all dystonia patients in need would not only improve their well-being dramatically, but also would be possible within the cost structures of most of our health care systems. Applying various strategies may be used for cost reductions. Increasing competition, especially from countries outside of Europe and North America, will certainly further reduce costs in the future.

## Data Availability

Raw data are available upon reasonable request.

## References

[CR1] Arzneimittel-Atlas IGES Institut GmbH, Berlin (2022) Germany/13.10.22/https://www.arzneimittel-atlas.de/arzneimittel/n04-antiparkinsonmittel/ausgaben/

[CR2] Brefel-Courbon C, Simonetta-Moreau M, Moré C, Rascol O, Clanet M, Montastruc JL, Lapeyre-Mestre M (2000) A pharmacoeconomic evaluation of botulinum toxin in the treatment of spasmodic torticollis. Clin Neuropharmacol 23:203–207 10.1097/00002826-200007000-00006 11020124

[CR3] Burbaud P, Ducerf C, Cugy E, Dubos JL, Muller F, Guehl D, Dehail P, Cugy D, Moore N, Lagueny A, Joseph PA (2011) Botulinum toxin treatment in neurological practice: how much does it really cost? A prospective cost-effectiveness study. J Neurol 258:1670–1675 10.1007/s00415-011-5998-9 21424611

[CR15] D-Statis (2022) 13.10.22. https://www.destatis.de/EN/Themes/Society-Environment/Population/Current-Population/_node.html

[CR4] Dodel RC, Kirchner A, Koehne-Volland R, Künig G, Ceballos-Baumann A, Naumann M, Brashear A, Richter HP, Szucs TD, Oertel WH (1997) Costs of treating dystonias and hemifacial spasm with botulinum toxin A. PharmacoEconomics 12:695–706 10.2165/00019053-199712060-00009 10175981

[CR5] Dressler D (2009) Routine use of Xeomin in patients previously treated with Botox: long term results. Eur J Neurol 16(Suppl 2):2–5 10.1111/j.1468-1331.2009.02877.x 20002739

[CR6] Dressler D, Adib Saberi F (2017) Economics of botulinum toxin therapy: influence of the abobotulinumtoxinA package size on the costs of botulinum toxin therapy. J Clin Mov Disord 4:6. 10.1186/s40734-017-0049-z 10.1186/s40734-017-0049-z PMC5406912 28465830

[CR7] Dressler D, Bigalke H (2017) Long-term stability of reconstituted incobotulinumtoxinA: how can we reduce costs of botulinum toxin therapy? J Neural Transm 124:1223–1225 10.1007/s00702-017-1767-y 28770389

[CR8] Dressler D, Johnson EA (2022) Botulinum toxin therapy: past, present and future developments. J Neural Transm 129:829–833 10.1007/s00702-022-02494-5 PMC9188496 35396965

[CR9] Dressler D, Mander G, Fink K (2012) Measuring the potency labelling of onabotulinumtoxinA (Botox(^®^)) and incobotulinumtoxinA (Xeomin (^®^)) in an LD50 assay. J Neural Transm 119:13–15 10.1007/s00702-011-0719-1 21971766

[CR10] Dressler D, Tacik P, Adib Saberi F (2014) Botulinum toxin therapy of cervical dystonia: comparing onabotulinumtoxinA (Botox(^®^)) and incobotulinumtoxinA (Xeomin (^®^)). J Neural Transm 121:29–31 10.1007/s00702-013-1076-z PMC3889804 23913131

[CR11] Dressler D, Altenmueller E, Bhidayasiri R, Bohlega S, Chana P, Chung TM, Frucht S, Garcia-Ruiz PJ, Kaelin A, Kaji R, Kanovsky P, Laskawi R, Micheli F, Orlova O, Relja M, Rosales R, Slawek J, Timerbaeva S, Warner TT, Adib Saberi F (2016) Strategies for treatment of dystonia. J Neural Transm 123:251–258 10.1007/s00702-015-1453-x 26370676

[CR12] Dressler D, Pan L, Bigalke H (2019) Comparing incobotulinumtoxinA (Xeomin^®^) and onabotulinumtoxinA (Botox^®^): identical potency labelling in the hemidiaphragm assay. J Neural Transm 125:1351–1354 10.1007/s00702-018-1897-x 29946929

[CR13] Dressler D, Altavista MC, Altenmueller E, Bhidayasiri R, Bohlega S, Chana P, Chung TM, Colosimo C, Fheodoroff K, Garcia-Ruiz PJ, Jeon B, Jin L, Kanovsky P, Milanov I, Micheli F, Orlova O, Pandey S, Pirtosek Z, Relja M, Rosales R, Sagástegui-Rodríguez JA, Shahidi GA, Timerbaeva S, Wan X, Walter U, Adib Saberi F (2021) Consensus guidelines for botulinum toxin therapy: general algorithms and dosing tables for dystonia and spasticity. J Neural Transm 128:321–335 10.1007/s00702-021-02312-4 PMC7969540 33635442

[CR14] Dressler D, Altenmüller E, Giess R, Krauss JK, Adib Saberi F (2022) The epidemiology of dystonia: the Hannover epidemiology study. 10.1007/s00415-022-11310-9. J Neurol (epub ahead) 10.1007/s00415-022-11310-9 PMC9618521 35948800

[CR16] French G, Bosch JD, Randall DR (2020) Retrospective review of dosing trends in botulinum toxin injections for the treatment of adductor spasmodic dysphonia in a long-term cohort. J Otolaryngol Head Neck Surg 49:4. 10.1186/s40463-020-0401-4 10.1186/s40463-020-0401-4 PMC6961226 31937363

[CR17] GKV-Spitzenverband (2022) 13.10.22/https:https://www.gkv-spitzenverband.de/service/zahlen_und_grafiken/gkv_kennzahlen/gkv_kennzahlen.jsp)

[CR18] Gudex CM, Hawthorne MR, Butler AG, Duffey PO (1997) Measuring patient benefit from botulinum toxin in the treatment of dystonia. Feasibility of cost-utility analysis. PharmacoEconomics 12:675–684 10.2165/00019053-199712060-00007 10175979

[CR19] Holden PK, Vokes DE, Taylor MB, Till JA, Crumley RL (2007) Long-term botulinum toxin dose consistency for treatment of adductor spasmodic dysphonia. Ann Otol Rhinol Laryngol 116:891–896 10.1177/000348940711601204 18217507

[CR20] Pan L, Bigalke H, Kopp B, Jin L, Dressler D (2019) Comparing lanbotulinumtoxinA (Hengli^®^) with onabotulinumtoxinA (Botox^®^) and incobotulinumtoxinA (Xeomin^®^) in the mouse hemidiaphragm assay. J Neural Transm 126:1625–1629 10.1007/s00702-019-02100-1 31707463

[CR21] Peter G, Peterson Foundation (2022) How much does the United States spend on prescriptiom drugs compared to other countries? https://www.pgpf.org/blog/2022/11/how-much-does-the-united-states-spend-on-prescription-drugs-compared-to-other-countries. generated 07.11.22, retrieved 10.11.23)

